# Incidence, predictors and prognostic impact of acute kidney injury in patients with COVID-19

**DOI:** 10.12669/pjms.38.6.6475

**Published:** 2022

**Authors:** Sahrai Saeed, Dominique Guerrot

**Affiliations:** 1 Sahrai Saeed, MD, PhD, FESC. Department of Heart Disease, Haukeland University Hospital, Bergen, Norway; 2 Dominique Guerrot, MD, PhD. Service de Néphrologie & INSERM U1096, CHU Hôpitaux de Rouen, Rouen, France

**Keywords:** Acute kidney injury, COVID-19, Post-acute COVID-19 sequelae, Renal function

In patients with severe coronavirus disease 2019 (COVID-19), acute kidney injury (AKI) is common (24-57%), particularly in those treated in intensive care unit (ICU) (61-78%), and is associated with poor outcomes.^1^-^3^ The pathogenesis of AKI in this setting is multifactorial. Endothelial dysfunction, microvascular thrombi and kidney inflammation are considered as major contributors to kidney lesions and decreased kidney function related to severe SARS-CoV-2 infection.^4^ Higher age, obesity and pre-existing comorbid conditions, among others, immunosuppression, diabetes, hypertension, chronic kidney and lung disease, chronic systemic inflammatory disease and cancer are known risk factors for COVID-19 severity and development of organ damage during index hospitalization. Patients with severe COVID-19 who develop AKI have greater need for dialysis compared with AKI patients without COVID-19.^3^ Furthermore, recent studies have shown that COVID-19-induced AKI was associated with a greater percentage decline in estimated glomerular filtration rate (eGFR) 6-12 months after discharge compared with AKI patients without COVID-19.^3^,^5^

In a recent edition of the journal, (Pak J Med Sci) Anees et al.^6^, reported on the frequency and risk factors for AKI in COVID-19 patients admitted to ICU in Farooq Hospital, West Wood Branch, Lahore. The study was retrospective by design and included 176 patients (78.4% males and mean age 51.3 years). The mean duration of hospital stay was eight days (range 1-20). AKI was found in half of patients (51%). Although the mortality rates in the entire study population was low (5.1%), it was twice as common in AKI than No-AKI patients (3.4 vs 1.7%). In univariate logistic regression analyses, AKI was associated with higher age, prolonged hospital stay, diabetes, hypoxemia, leukocytosis and lymphopenia, hypoalbuminemia and elevated C-reactive protein and D-dimers, all well-known prognosticators in COVID-19. The presence of symptoms was also associated with a 6.6-fold increased risk of AKI. Presumably all patients had severe symptoms as they were admitted to the ICU. Furthermore, their multivariable-adjusted model identified hypoxemia, elevated inflammatory markers and prolonged hospital stay as predictors of AKI. However, it would be better to present a fully-adjusted model, as well as the criteria for entering the candidate covariates in the multivariate model. For comparison a large retrospective study from the US also explored the incidence and grade of AKI and impact on mortality in 1545 COVID-19 patients.^7^ Of note, the definition of AKI and the exclusion criteria, which are major issues when comparing studies on AKI, were similar to those defined by Anees et al.^6^ (pregnancy and pre-existing chronic kidney disease). The authors found a 39% (n=608) incidence of AKI (294 [n=48.4%] with Grade-I, 185 [n=30.4%] Grade-II, and 129 [n=21.2%] Grade-III), and with a mortality rate of 58.2% (n=354). Among patients with AKI, 42.6% (n=259) were admitted to the ICU. AKI was associated with age, hypertension, diabetes, congestive heart failure, pre-existing chronic kidney disease, hepatitis C and duration of hospital stay.^7^ The overall mortality rate was 28.6% (58.2% in AKI vs. 9.4 in No-AKI group, p<0.001). The higher mortality rates in this particular study may be explained by the higher age (mean age 62.6 years) and higher burden of cardiovascular disease compared with the study of Anees et al.^6^

However, although the incidence and clinical predictors of AKI during acute phase of COVID-19, and impact on prognosis are now well-established, it is important to direct focus towards those patients who survive severe COVID-19 and develop post-acute sequelae. These are defined as signs and symptoms lasting beyond 30 days after initial infection with SARS-CoV-2 virus. This involves pulmonary and extra-pulmonary organ manifestations, such as incident diabetes, arterial damage (increase pulse wave velocity and endothelial dysfunction demonstrated by reduced brachial flow-mediated dilatation) and subclinical myocardial dysfunction assessed by speckle-tracking echocardiography or cardiac magnetic resonance imaging with and without persistent myocardial inflammation and edema, elevated blood pressure and resting heart rate and anemia (Fig.1).^8^-^11^ In addition, there is now growing body of evidence suggesting that beyond the acute illness, and independent of pre-existing chronic renal disease, a decline in renal function six to 12 months after the index hospitalization for COVID-19 may also occur, reflecting post-acute COVID-19 outcomes.^12^ Finally, in nearly one-third of COVID-19 survivors, kidney function may not recover to baseline by hospital discharge.^2^ This underscores the importance of larger, prospective studies in the future to investigate the mechanisms of AKI and persistent kidney function impairment.

**Fig.1:**
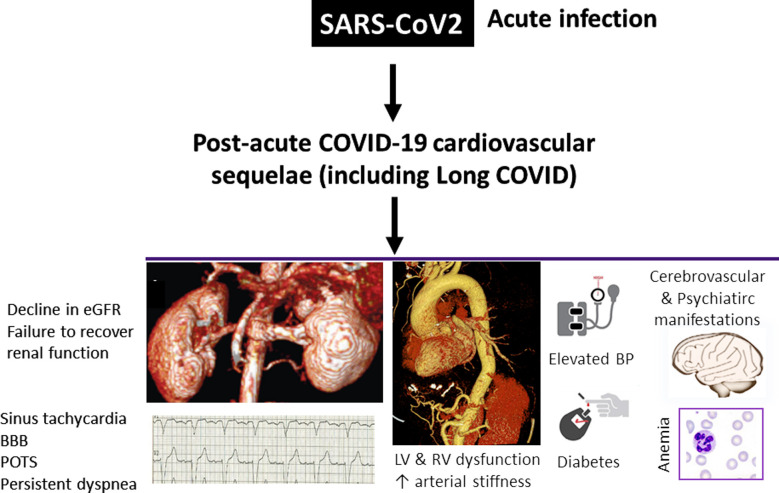
Common extrapulmonary manifestations in post-acute COVID-19 syndrome. **BBB,** bundle branch block; **BP,** blood pressure; **eGFR,** estimated glomerular filtration rate; **LV,** left ventricular; **POTS,** postural orthostatic tachycardia syndrome; **RV,** right ventricular.

## CONCLUSION

The incidence and clinical predictors of AKI during acute phase of COVID-19, and impact on prognosis are well-established. Future studies should direct their attention towards the late complications, particularly decline in renal function as post-acute COVID-19 syndrome sequelae, also referred to as long COVID. These late clinical outcomes require careful medical attention with earlier and more frequent follow-up strategies to avoid long-term cardiovascular complications.

### Authors’ Contribution:

**SS** prepared the first draft of the article which was edited by **DG**.

Both authors approved the final submission.
